# Supplementary material for: Chromothripsis during telomere crisis is independent of NHEJ, and consistent with a replicative origin
Source: Genome Res. 2019 May;29(5):737–49. doi: 10.1101/gr.240705.118 (PMC6499312; doi:10.1101/gr.240705.118)
Supplement: Supplemental Material [file supp_gr.240705.118_Supplemental_file_1.zip › contigs/annotated_contigs/DB86/contig.2.DB86_length_451_mean_cov_7.24168514412.docx]

**DB86_length_451_mean_cov_7.24168514412**

```
TATTGGCAGCCCCTAGGAAGAGAGAAGCTCGCCTCACATGAGCACAGAACTACTCTTCTTTCCTTGTCTTCCCACGTCCCATTGTGTCC
 >chr5:177857265-177857517 - E=4e-134
TTTCCAGGGGACTATGAGCCCCAGGATGGAATTGTGCAACTGGTGCCATTGATGTCCTTAGGAAATCGGAACCCGTTTCTGGAATTCTT

GACAGTGAGTGGCAGAATTCCAGTGGGCAGAGACCCAGGAACCTGGGGCCCCAGTGAAGTCACCTGTGTGTCT|T|GAAGTCAGGAGTT
                                                                          >chr5:1774283
TGAGACTAGCCTGGCCAACATGGTGAAACCCTGTCTTTACTAAAAATACAAAAATTAGCCGGGTCTGGTGGCAGGCACCTGTAGTCCCA
14-177428514 - E=9e-88
GCTACTCAGGAGGCTGAGGGAGGCAAATCACTGGACCACAGGAGGCAGAAGTTGCGGTGAGCCCAGACGGTGCCCCTGCACCCTAGCCT

GGGCAACA
```
